# Critical Design Parameters of Tantalum-Based Comb Structures to Manipulate Mammalian Cell Morphology

**DOI:** 10.3390/ma18092099

**Published:** 2025-05-03

**Authors:** Hassan I. Moussa, Megan Logan, Ali Eskandari, D. Moira Glerum, Marc G. Aucoin, Ting Y. Tsui

**Affiliations:** 1 Department of Chemical Engineering, University of Waterloo, Waterloo, ON N2L 3G1, Canada; h2moussa@uwaterloo.ca (H.I.M.); megan.k.logan@gmail.com (M.L.); ali.eskandari@uwaterloo.ca (A.E.); marc.aucoin@uwaterloo.ca (M.G.A.); 2 Waterloo Institute for Nanotechnology, University of Waterloo, Waterloo, ON N2L 3G1, Canada; 3 Department of Biology, University of Waterloo, Waterloo, ON N2L 3G1, Canada

**Keywords:** tantalum, mammalian cells, Vero cells, silicon oxide, adhesion, morphology

## Abstract

Mammalian tissues and cells often orient naturally in specific patterns to function effectively. This cellular alignment is influenced by the chemical nature and topographic features of the extracellular matrix. In implants, a range of different materials have been used in vivo. Of those, tantalum and its alloys are promising materials, especially in orthopedic implant applications. Previous studies have demonstrated that nano- and micro-scale surface features, such as symmetric comb structures, can significantly affect cell behavior and alignment. However, patterning need not be restricted to symmetric geometries, and there remains a gap in knowledge regarding how cells respond to asymmetric comb structures, where the widths of the trenches and lines in the comb differ. This study aims to address this gap by examining how Vero cells (cells derived from an African green monkey) respond when applied to tantalum and tantalum/silicon oxide asymmetric comb structures having fixed trench widths of 1 μm and line widths ranging from 3 μm to 50 μm. We also look at the cell responses on inverted patterns, where the line widths were fixed at 1 μm while trench widths varied. The orientation and morphology of the adherent cells were analyzed using fluorescence confocal microscopy and scanning electron microscopy. Our results indicate that the widths of the trenches and lines are important design parameters influencing cell behavior on asymmetric comb structures. Furthermore, the ability to manipulate cell morphology using these structures decreased when parts of the tantalum lines were replaced with silicon oxide.

## 1. Introduction

Biological cells and tissues often require a specific arrangement to function properly. These organized cell structures can be found in different organs, such as muscles [1], intestines [2], eyes [3], and skin [4]. The chemical composition and structure of the microenvironment to which cells attach [5], such as the naturally produced extracellular matrix (ECM) within the body, or artificially engineered surfaces, can influence the organization of these tissue structures.

There are several approaches that can be used to manipulate cell functions on engineered surfaces, such as surface topography, surface composition, biochemical patterning, or dynamic substrates [6]. The first technique involves using surface topography to induce desirable cell behavior through a mechanotransduction approach. These topographic features are created with materials like polymers [7,8,9,10,11,12,13,14,15], metals [16,17,18,19,20,21], or silicon [22]. Apart from using monolithic micro- to nano-topographic features to control cell behavior, another method is through surface composition [20,23,24,25,26,27,28] and patterning with different materials [24,25,27] or functionalized groups [23,28]. Since different materials may have varying surface energies, charges, or adsorption characteristics, proteins and other organic molecules may preferentially adhere to or adsorb on specific materials or locations on the engineered surface. Moussa et al. have shown that patterned surface composites consisting of silicon oxide (SiO_2_) and tungsten can be used to modify the adherence characteristics of African green monkey (Vero) [29] and human dermal fibroblast (GM5565) cells [30]. Their work demonstrated that cells preferentially adhere to tungsten rather than SiO_2_ and change their shape to maximize contact area with the metal. Biochemical patterning has also been shown to be a successful technique for controlling cell morphology and function [31,32,33,34,35,36]. For example, Poudel et al. [36] micropatterned collagen-I, PLL-g-PEG, and retinoic acid on polystyrene substrates. The patterned surface was used to control the morphology and outgrowth behavior of neuronal cells and their neurites. Their results showed that almost all of the cell nuclei were oriented within 10° of the pattern direction on substrates containing 5 μm and 10 μm wide protein lane structures. Cell functions can also be manipulated by using the dynamic substrate methods, where the stiffness of the engineered substrate or the properties of functional groups adsorbed on substrate surfaces are altered or controlled by external physical sources, such as light [37,38,39].

Using engineered surfaces to control cell morphology is of interest to biomedical applications, such as orthopedic implants and cell immobilization devices. For example, Rani et al. [20] utilized surface nanostructures to enhance protein adsorption and induce human hipbone osteoblast cell (pHOB) proliferation on titanium implants. Other artificial surface structures improve osteogenesis and the implant success rate [20,40,41]. However, a better understanding is needed to determine design parameters for controlling cell morphology and function. For example, is the line width more crucial than trench width, or are they equally important?

Recently, researchers [25,42] combined two engineering approaches to create a new engineered composite surface (comb structure) for manipulating Vero cells. The motivation behind this prior work stems from the idea that if cells preferentially adhere to Ta over SiO_2_, as demonstrated in [25], combining the topographically driven and dissimilar material approach may enhance the cell controlling capability of the engineered surface. This comb structure consists of alternating tantalum (Ta) trenches and silicon oxide (SiO_2_) lines of equal width. They demonstrated that about 90% of the Vero cell population on these surface composites aligns parallel to the line axes. Similar surface topography-induced cell alignment behavior was observed for GM5565 cells on the same structures [42]. It is unclear whether surface topography or the use of dissimilar materials contributed more to the manipulation of cells on these Ta-based composite structures. Additionally, previous works [18,25,27] only investigated Ta-based comb structures with identical line and trench widths (symmetric structures) to manipulate cells. It is uncertain whether these comb structures can effectively manipulate cell behaviors when the line and trench widths are not identical (asymmetrical structures). While a few prior works [7,36,43,44,45] investigated the influence of trench and line width ratios of asymmetrical structures on cells, the range of width parameters studied is limited, and the substrate was not a Ta-based material.

The current study examines how the design parameters of Ta-based asymmetrical comb structures, specifically the line and trench widths, affect the behavior of Vero cells [46]. These cells were placed on patterned substrate surfaces with either a fixed line width or trench width of 1 μm, while the other width parameter was varied between 3 μm and 50 μm. Fluorescence and electron microscopy were used to analyze the shape and position of cells in relation to the patterned axes on the comb structure. This study also explores how the material and composition of the substrate affected cell behavior by comparing their responses on (i) monolithic Ta surfaces and (ii) Ta/SiO_2_ composite substrates. In the composite substrates, the trenches were coated with a Ta film, while the top surfaces of the lines consisted of exposed SiO_2_. Tantalum and its alloys are promising materials in orthopedic implant applications due to their excellent mechanical strength and biocompatibility, which promote new bone formation [25,47,48,49,50,51,52]. The tantalum-weighted surface energy of ~2.42 J/m^2^ [53] is larger than that of titanium of ~2.0 J/m^2^ [53]. Higher surface energy generally leads to better cellular adhesion to surfaces [54]. Hallab et al. [54] have shown that the cellular adhesion strength of 3T3MC fibroblasts on tantalum is higher than that of glass and polytetrafluoroethylene specimens. Additionally, prior work [55] has demonstrated that human osteoblasts perform better in cellular adhesion, growth, and differentiation on porous tantalum compared to titanium control specimens. The findings of this manuscript show that cells on Ta-based asymmetric comb structures are less likely to line up parallel to the pattern axes if the trench or line widths are wider than 3 μm. The results suggest that both pattern design factors are important for manipulating cells. Surprisingly, using multiple materials on the engineered surfaces did not improve cell alignment preference. Replacing the Ta-line features with SiO_2_ reduced the number of cells aligned to the axes, reducing the ability to control cell orientation.

## 2. Materials and Methods

### 2.1. Test Structure Fabrication

**Figure 1:**
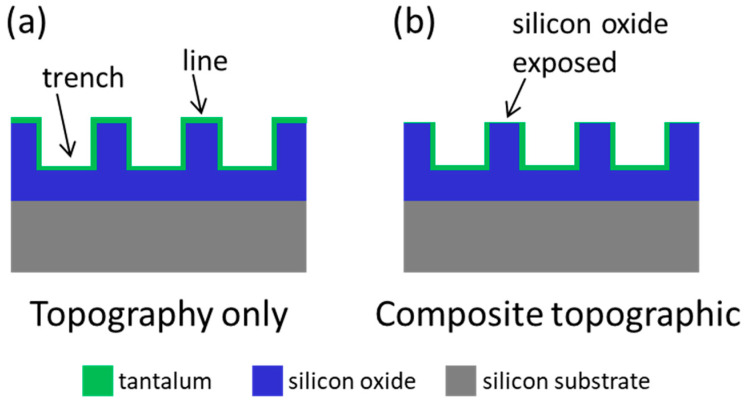
Schematic drawings illustrating the cross-sectional profile of the two sample configurations show that (**a**) the entire surface is covered with tantalum and that (**b**) silicon oxide is exposed at the top of the lines while the trench bottoms and sidewalls are coated with tantalum.

The substrate specimens tested in this study were provided by Versum Materials, LLC (Tempe, AZ, USA), and fabricated using photolithography and chemical etching processes, as detailed elsewhere [18,25]. Briefly, test chips were created on 200 mm silicon wafers. Each chip contains all test structures. During the fabrication process, all test structures on each chip were processed simultaneously under identical processing conditions. This ensures that all structures on a chip experience the same process condition. After the trenches were created on the substrates, they were coated with a Ta seed layer and filled with copper (Cu). The excess Cu was removed by utilizing the chemical–mechanical process (CMP). Different CMP endpoints and Cu stripping techniques created two types of substrate surfaces, as schematically illustrated in Figure 1. Figure 1a shows that a thin coating of Ta (green) was applied to the trench sidewall and the surfaces of the line and trench. The composite substrates (Figure 1b) contain a line top surface with exposed SiO_2_ (blue). Asymmetric comb patterns were fabricated with different combinations of trench and line widths ranging from 1 μm to 50 μm, as detailed in Table 1. This included line width to trench width ratios from 0.02 to 50.

### 2.2. Cell Culture and Deposition

Mammalian cells (Vero) were obtained from the American Type Culture Collection (ATCC, Manassas, VA, USA). Cells were prepared using the same procedure described in [18,25]. Briefly, cells were cultured in tissue-culture-treated 75 cm^2^ flasks (Corning Falcon, VA, USA). The culture media contained MEM/F12 media (Corning, New York, NY, USA), 10% (*v*/*v*) of Gibco™ fetal bovine serum (FBS, Thermo Fisher Scientific, Waltham, MA, USA), and 4 mM of L-glutamine (Sigma-Aldrich, St. Louis, MO, USA). Before seeding cells, the substrates were submerged in ethanol (70%) for 30 s, air-dried, and rinsed with Dulbecco’s phosphate-buffered saline (D-PBS). The cell concentration seeded on the substrates was targeted at ~0.5 × 10^5^ cells/mL, and they were incubated in 6-well tissue culture plates (Nunc, Thermo Scientific, Copenhagen, Denmark) at 37 °C for 24 h. Researchers [27,44] have shown that cells require ~24 h to complete the alignment process on engineered structures. Since all the test structures are located on a single chip with different geometric features, they were exposed to the same cell seeding conditions simultaneously.

### 2.3. Cell Fixation, Microscopy, and Measurement Parameters of Cell Alignment

**Figure 2:**
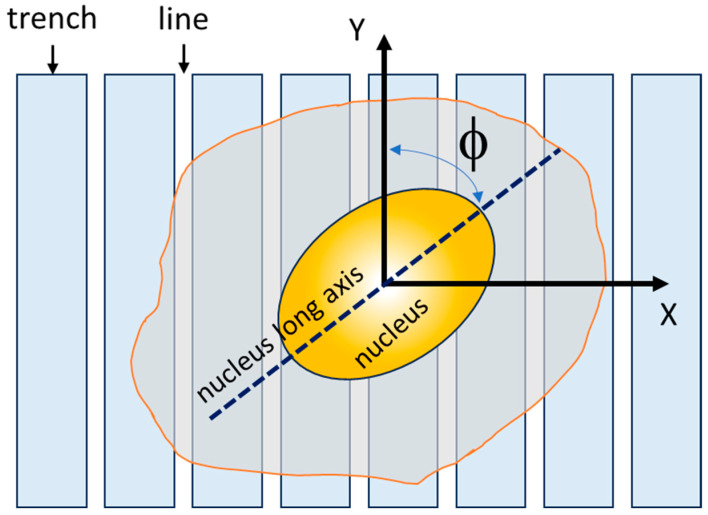
This figure shows a schematic illustration of the angular displacement (ϕ) between the cell nucleus’s long axis and the line axes used to quantify cell alignment.

After 24 h of incubation, adherent cells on the substrates were rinsed with D-PBS and fixed with 4% methanol-free formaldehyde (Sigma-Aldrich, Oakville, ON, Canada) for 1 h. Specimens were sequentially dehydrated with 50%, 75%, 95%, and 100% (*v*/*v*) ethanol solutions for 10 min for each concentration. A Zeiss 1550 field-emission scanning electron microscope (SEM) (Carl Zeiss AG, Oberkochen, Germany) operating at 7 kV was used to characterize the morphology and alignment of the cells. None of the specimens were coated with a conductive layer to avoid generating image artifacts. The angular displacement (ϕ) between the long axis of an adherent cell nucleus with respect to comb structure was measured, as illustrated in Figure 2.

### 2.4. Cell Staining

Substrates with fixed adherent cells were permeabilized with 2 mL of 0.1% Triton-X (Sigma-Aldrich, St. Louis, MO, USA) for 5 min and rinsed with PBS. F-actin microfilaments were exposed to Phalloidin-iFluor 647 Reagent (ab176759, Abcam Inc., Cambridge, MA, USA) for 45 min prior to being rinsed with PBS. The deoxyribonucleic acid (DNA) molecules were stained 4′,6-diamidino-2-phenylin-dole (DAPI) diluted in PBS (1:25,000) (Life Technologies, Burlington, ON, Canada) for 5 min followed with three 2 mL PBS rinses. Prepared specimens were kept at 4 °C. Stained cells were inspected with a Leica TCS SP5 confocal microscope (Leica, Wetzlar, Germany) at the University of Guelph (Guelph, ON, Canada).

## 3. Results and Discussion

### 3.1. Cell Alignment on Ta Topographic Structures

In the first part of this study, we investigated how cells behaved on comb-shaped structures covered with a blanket layer of Ta (Figure 1a). Two groups of substrate patterns were created to investigate the effect of line and trench widths. The first set of substrates had parallel line comb structures with a fixed line width of 1 μm, with the width of the trenches varying from 3 μm to 50 μm. The second set of substrates had an inverted comb pattern with a fixed trench width of 1 μm, with varying line widths. A detailed sample list is shown above in Table 1. This range of line-to-trench width ratios (from 0.02 to 50) is more extensive than prior studies [8,15]. Vero cells were incubated on these asymmetric structures for 24 h, and their morphology was analyzed using top-down fluorescence confocal microscopy and field-emission SEM.

**Figure 3:**
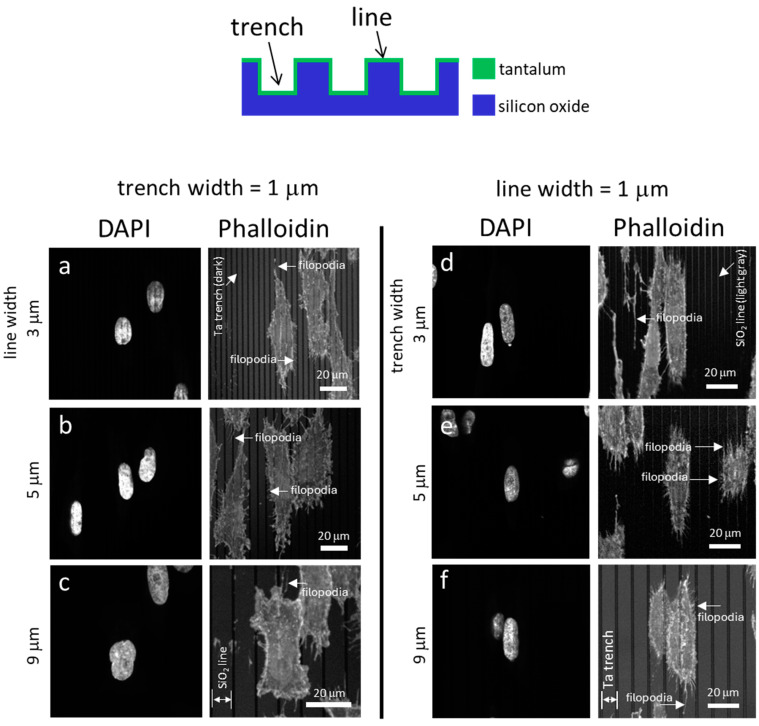
Typical fluorescence confocal micrographs show cells adhered to patterned comb structures. The substrate surfaces are covered with a uniform layer of Ta thin film. The left panel (**a**–**c**) consists of images of cells on substrates with a fixed trench width of 1 μm. Adherent cells on substrates patterned with lines with widths of 1 μm are shown in (**d**–**f**). Scale bars correspond to 20 μm. The DNA molecules were stained with DAPI. Phalloidin fluorescence stains were used to label actin microfilaments.

**Figure 4:**
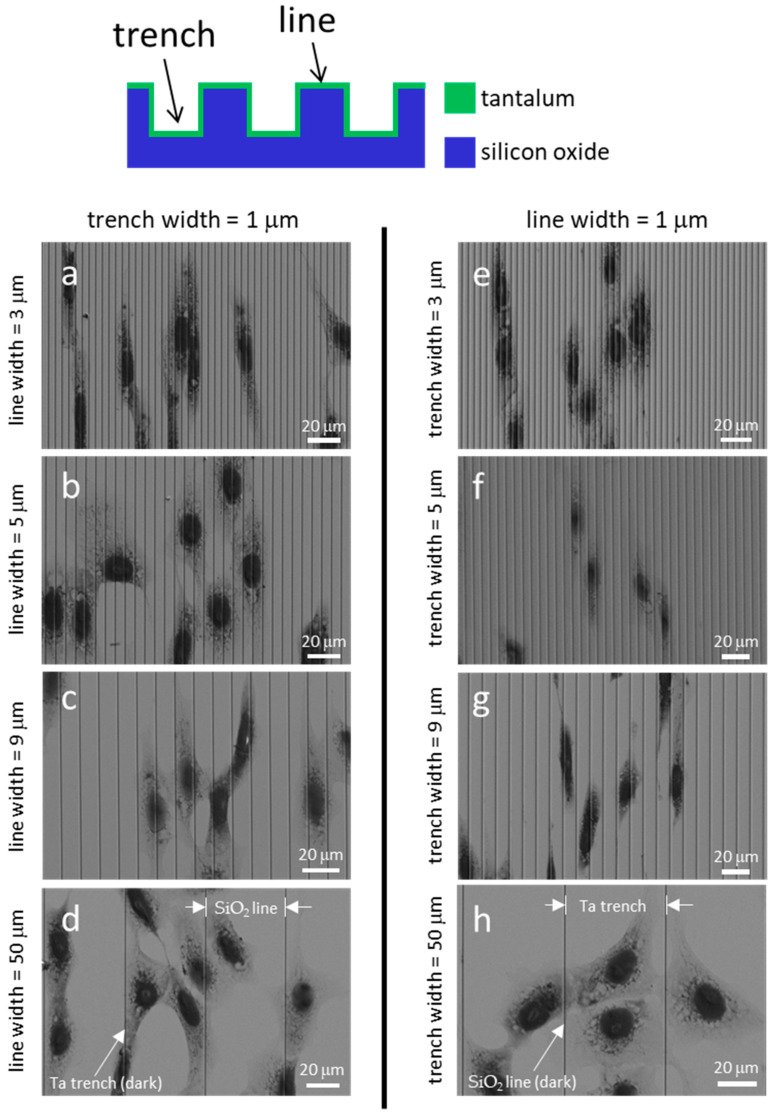
Typical scanning electron micrographs revealed cells attached to various pattern structures. The substrate surfaces are covered with a blanket layer of Ta thin films. Figures (**a**–**d**) show structures with a fixed trench width of 1 μm. Cells on structures with fixed line widths of 1 μm are shown in (**e**–**h**). Patterned lines are oriented vertically in the images.

Figure 3 displays typical fluorescence confocal micrographs of adherent cells on the Ta-coated comb structures. The DNA molecules were stained with DAPI, while the F-actin microfilaments were labeled with the phalloidin-iFluor Reagent. Figure 3a, Figure 3b, and Figure 3c show images of cells on structures consisting of trenches with fixed widths of 1 μm and line widths of 3 μm, 5 μm, and 9 μm, respectively. Micrographs of cells on the inverted patterns containing a fixed line width of 1 μm and varying trench widths are shown in Figure 3d–f. The images show that most cells and nuclei inspected are elongated and have various degrees of preference for aligning with the line axes. Filopodia protrude from cells in all directions, with a longer length along the line axes than in the lateral direction. The adherent cells were also examined using high-resolution SEM (Figure 4). The micrographs in the left panels (Figure 4a, Figure 4b, Figure 4c and Figure 4d) show adherent cells on comb structures with fixed trench widths of 1 μm and line widths of 3 μm, 5 μm, 9 μm, and 50 μm, respectively. The results indicate that elongated cells align with the long axes of the comb structures on substrates with line widths of 3 μm, 5 μm, and 9 μm. However, cells did not show a strong alignment preference on substrates with a line width of 50 μm (Figure 4d). Micrographs in Figure 4e–h display cells cultured on substrates with inverted patterns and a fixed line width of 1 μm. Figure 4e, Figure 4f and Figure 4g shows that adherent cells tend to align parallel to the line axes on comb structures consisting of 3 μm, 5 μm, or 9 μm trenches. However, Figure 4h shows that adherent cells on a pattern with a trench width of 50 μm are more circular and more randomly oriented when compared with cells on a pattern with narrower trenches (see Figure 4e–g). These microscopy results suggest that line and trench widths are critical design parameters and can influence cell morphology on Ta-based asymmetrically patterned substrates. Increasing the width of the line or trench to 50 μm while maintaining the other pattern feature at 1 μm decreased the aligned cell population.

**Figure 5:**
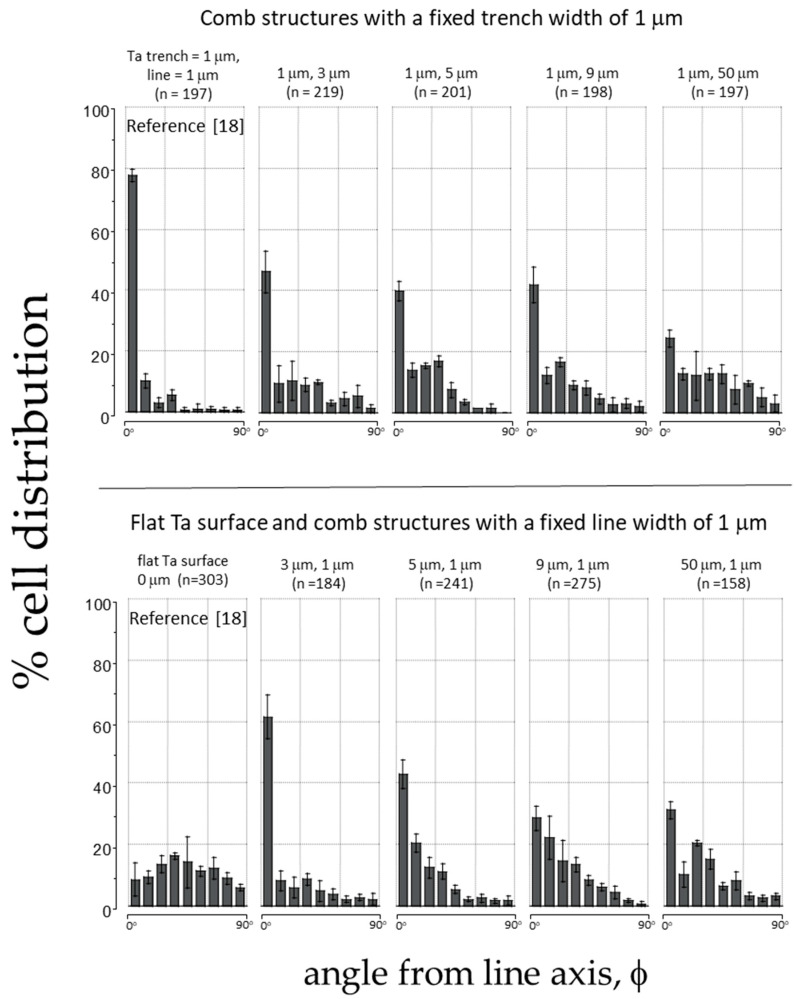
This figure shows the percent cell distribution of cell orientation relative to the line axes (ϕ) after incubation on substrates with various trench and line widths. Each bin corresponds to the cell population within ±10° angular range. The number of cells (n) inspected for each comb structure is also included. Prior results on the flat Ta surface [18] and comb structure [18] with equal lines and trench widths of 1 μm are also included in this figure.

After visualizing cell morphology using microscopy techniques, we quantified how the cells aligned on patterned substrates by measuring the angle (ϕ) between the nucleus’s long axis and comb structure axis, as shown in Figure 2. Figure 5 displays the percentage of cells oriented at different angles on the comb structures and the total number of cells (n) examined in the corresponding pattern. Each plot in Figure 5 shows bins representing the percentage of the cell population within a 10° range. For instance, the second bin in each plot includes results from cells with nuclei orientations ranging from −11° to −20° and 11° to 20° relative to the line axes. The data spread in the figure represent one standard deviation from three random groups of cells. The alignment results on structures with a fixed trench width of 1 μm and increasing line widths of 3 μm, 5 μm, 9 μm, and 50 μm are presented in the upper row of Figure 5. Additionally, results from our previous study [18] on non-topographic flat surfaces and symmetric comb structures with equal trench and line widths of 1 μm are included in this figure as a comparison. The results indicate that cells were less likely to align parallel to the axes when adhering to structures with increased line widths. For example, on the symmetric comb structure with equal line and trench widths of 1 μm, most cells (approximately 78% of the cell population) were aligned within ± 10° to the line axes (bin 1). In contrast, smaller proportions of cells favored alignment parallel to the lines on substrates with line widths greater than 3 μm.

The lower panels of Figure 5 show how cells oriented on surfaces with patterns consisting of 1 μm lines and varying trench widths. The results indicate that, as the trench width increases, the cells’ tendency to align parallel to the axes decreases. This suggests that the effectiveness of using a Ta-based comb feature to control cell behavior depends on the pattern design and is limited by the larger dimension of the two design parameters (either the trench or line width). When one of the width parameters exceeds 3 μm, the likelihood of cells aligning on these asymmetric comb structures decreases. These results agree with the cell shape seen in the SEM images in Figure 4, underscoring the importance of comb structure design in controlling cell shape.

### 3.2. Cell Alignment on Ta/SiO_2_ Composite Surface Structures

The results of cell orientation discussed in the previous section have helped us understand how monolithic Ta topographic features affect cell behavior. However, as composite materials are increasingly used in biomedical devices like implants, it is essential to investigate the combined impact of surface topography and different surface materials on cell behavior. While previous studies [7,9,14,18,23,27,36,56,57] have shown that surface topography and dissimilar surface materials can individually manipulate cell function, it is unclear whether these two approaches work together to produce an additive effect when applied to the same structure. To explore this, composite substrates containing dissimilar materials (Ta and SiO_2_) with the same topographic features tested in Section 3.1 were fabricated. Adherent cells on these composite surfaces were influenced by the topographic features and the dissimilar materials simultaneously. By comparing the cell orientation results obtained from monolithic and composite substrates, we determined the extent to which each approach contributed to cell manipulation.

**Figure 6:**
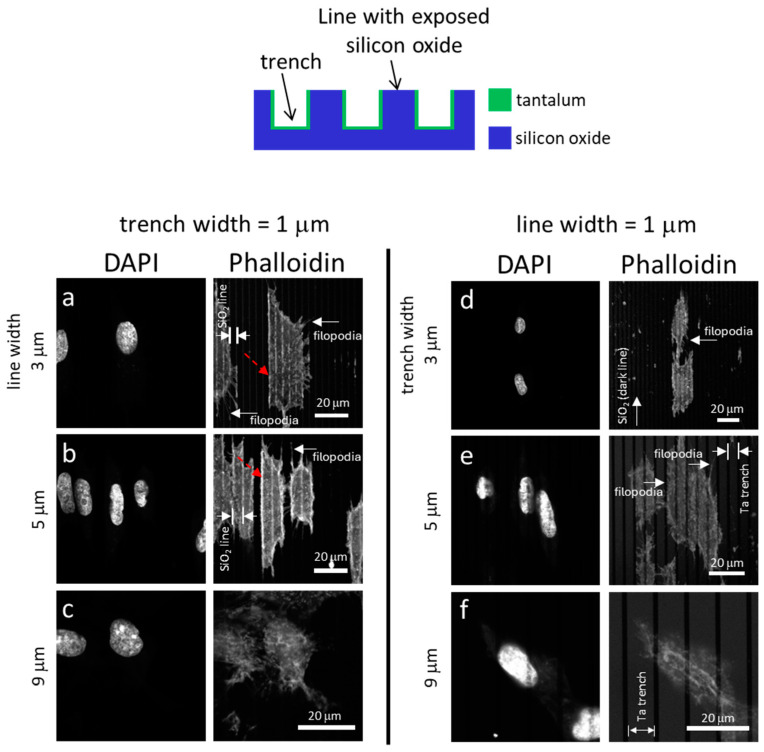
Typical fluorescence confocal micrographs show adherent cells on patterned structures. The substrate surfaces contain Ta-coated trenches and silicon oxide lines. Images of adherent cells on substrates with fixed trench widths of 1 μm are shown in (**a**–**c**). Cells on patterns with fixed line widths of 1 μm are displayed on the left panel (**d**–**f**). Scale bars correspond to 20 μm. The DNA molecules were stained with DAPI. Phalloidin fluorescence stains were used to label actin microfilaments.

Fluorescent confocal microscopy first assessed adherent cell morphology on Ta/SiO_2_ substrates and their images are displayed in Figure 6. Figure 6a, Figure 6b, and Figure 6c show representative images of cells on structures with fixed trench widths of 1 μm and line widths of 3 μm, 5 μm, and 9 μm, respectively. It was observed that structures with 3 μm and 5 μm line widths favored cell orientation parallel to the axes, but less so on structures with 9 μm lines. In addition, some cells tended to form straight edges positioned along the Ta trenches, as highlighted by the dashed arrows in Figure 6a,b. One possible explanation for this behavior is the selective adhesion of cells to Ta over SiO_2_. The trench walls were coated with tantalum, while the line surface consisted of SiO_2_. Cells changed their shape to maximize contact with the preferred material, which was tantalum in this case. Hallab et al. [54] have shown that the adhesion shear strength of the fibroblast cell (3T3) on a Ta surface is 407.10 dynes/cm^2^, which is greater than that on glass (Corning™ tissue culture Petri dishes) of 254 dynes/cm^2^. They also measured the total surface energy of the two materials with values of 100.59 ergs/cm^2^ and 69.78 ergs/cm^2^, respectively. Generally, materials with a larger surface energy tend to exhibit better cell adhesion. The observations reported in Figure 6a,c are consistent with the findings in the literature. Cell behavior on inverted patterns is shown in the right column of the images in Figure 6d–f. The micrographs show cell alignment on structures with 3 μm and 5 μm wide trenches, but less organization on structures with 9 μm trench widths.

**Figure 7:**
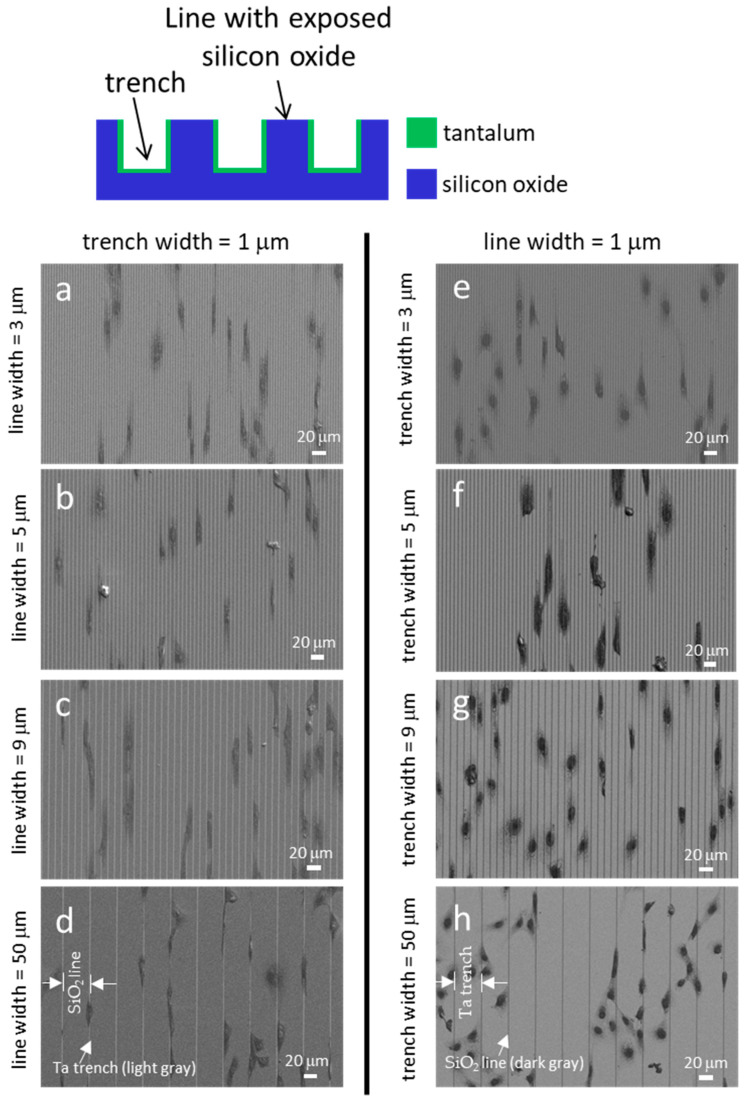
Typical scanning electron micrographs reveal adherent cells on engineered structures. Figures (**a**–**d**) show comb structures consisting of a fixed trench width of 1 μm with increasing line widths of 3 μm, 5 μm, 9 μm, and 50 μm, respectively. Cells on inverted design pattern structures containing line widths of 1 μm and trench widths of 3 μm, 5 μm, 9 μm, and 50 μm are displayed in (**e**–**h**), respectively. Light gray features are trenches, while dark gray areas are lines. Micrographs show cells are less likely to orient with pattern axes with trench widths. Scale bars represent 20 μm.

SEM micrographs of cells are displayed in Figure 7. The light gray regions in these images correspond to Ta-coated trenches, while the dark gray areas represent SiO_2_ lines. Figure 7a, Figure 7b, Figure 7c, and Figure 7d display the SEM micrographs of cells on comb structures with 1 μm wide Ta trenches and SiO_2_ line widths of 3 μm, 5 μm, 9 μm, and 50 μm, respectively. The images demonstrate that the cells elongated and oriented parallel to the line axes on these structures. Figure 7e, Figure 7f, Figure 7g, and Figure 7h show the morphology of cells on the composite substrate with inverted patterns. These comb structures consisted of 1 μm SiO_2_ lines and Ta trenches with widths of 3 μm, 5 μm, 9 μm, and 50 μm, respectively. The micrographs reveal that the adherent cells strongly favored orienting parallel to the line axes on structures with Ta trench widths of 3 μm, 5 μm, and 9 μm. However, in Figure 7h, it is evident that cells were less likely to align to a specific orientation on substrates consisting of 50 μm trenches.

The cell morphology on the monolithic Ta substrate (Figure 4d) and composite substrate (Figure 7d), both patterned with trench-to-line widths of 1:50, exhibited interesting differences. Despite having the same alternating parallel line/trench geometric features, the composite substrates contained lines with exposed SiO_2_ top surfaces, as depicted in Figure 1. In Figure 4d, cells on the monolithic Ta substrate, influenced solely by the topographic effect, were seen to spread on both lines and trenches without any specific segregation. In contrast, cells in Figure 7d attach and elongate along the 1 μm Ta trenches on the composite substrate. The differential cell response can be attributed to selective cell adhesion on Ta. Furthermore, the absence of segregated cells at SiO_2_ lines in Figure 7h suggests that cells favor Ta over SiO_2_ surfaces. This behavior aligns with findings from Moussa et al. [27], who observed similar cell segregation and shape-changing behavior on isolated 180 nm wide tungsten lines embedded in a SiO_2_ substrate. Additionally, Pudel et al. [36] reported preferential attachment and elongation of human neuroblastoma cells (SH-SY5Y) on 5 μm collagen-I lines micro-patterned on tissue culture polystyrene substrates.

**Figure 8:**
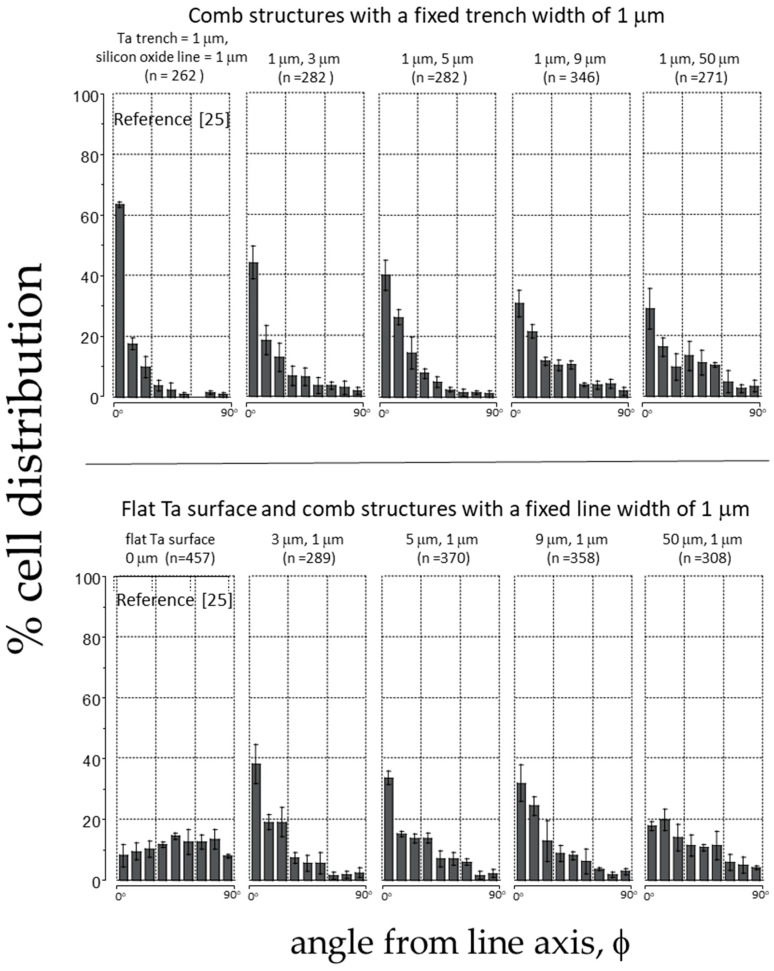
The figure shows the percent cell distribution of cell orientation relative to the line axes (ϕ) after incubation on substrates with various trench and line widths. Each bin corresponds to the cell population within a ± 10-degree angular range. The trench bottom and sidewalls are covered with tantalum, while the line top surface is SiO_2_ [25].

The data presented in Figure 8 shows how cells aligned on asymmetric comb structures made of Ta and SiO_2_. The figure includes data from previous research [25] on a Ta surface without topographical features and a comb structure consisting of alternating Ta trench/SiO_2_ lines with equal widths of 1 μm. The top panels of the figure display the alignment results of cells on structures with fixed Ta trench widths of 1 μm and SiO_2_ lines with widths ranging from 3 μm to 50 μm. The results indicate that the highest percentage (approximately 62%) of cells align within ±10° of the line axes on structures with equal line and trench widths of 1 μm. However, the tendency for alignment decreases as the line widths increase with no more than ~30% of cells aligning to comb structures containing lines 50 μm wide. The bottom panel of Figure 8 shows the cell performance on surfaces with inverted patterns, where the line width is maintained at 1 μm. The results reveal that the aligned cell population decreases with an increase in the trench width, consistent with the observations in the micrograph images presented in Figure 7e–h. This observation supports the findings reported in Section 3.1, where cells are less likely to organize parallel to the axes when one of the width parameters increases (either trench or line widths), even when one of the feature sizes is maintained at 1 μm.

### 3.3. Pattern and Material-Dependent Cell Alignment Degradation

**Figure 9:**
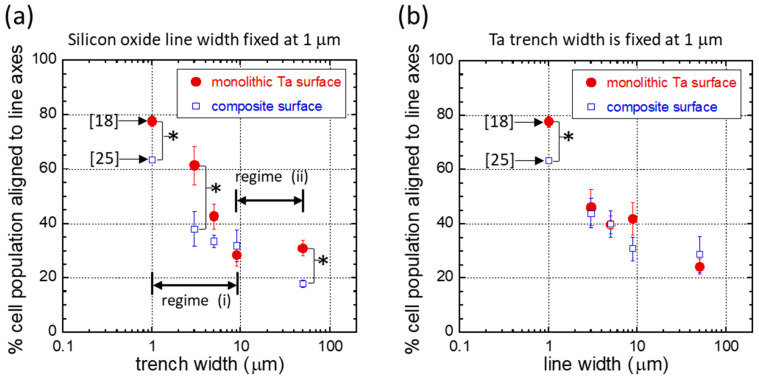
Plots of the percent population of cells oriented within ± 10° of the line axes for comb structures consisting of (**a**) a fixed line width of 1 μm and (**b**) a fixed trench width of 1 μm. The solid red circles represent the data collected from the monolithic Ta comb structures. Open squares denote the results of cells adhered to tantalum/SiO_2_ composite surfaces. Results from references [18,25] are included in these plots as a comparison. Patterns highlighted with (*) indicate a significant difference in cell alignment induced by the two types of substrates.

Line and trench widths, as well as material properties, affect cell orientation. To further quantify the interplay between material design and comb pattern geometries on cell behavior, the population of aligned cells (cells oriented within ± 10° of the line axes) on different substrates and patterns are displayed in Figure 9. Figure 9a and Figure 9b illustrate the tendency for cells to align on structures with a fixed line width and trench width of 1 μm, respectively. The red solid circles represent measurements from comb structures made of monolithic Ta surfaces, showing how the cells respond to topographic features alone. The blue open squares show cell behavior on composite surfaces, consisting of alternating Ta trenches and SiO_2_ lines. These results indicate that the topographic features and the composition of comb structures influence how cells adhere to them. Figure 9 also includes data from prior studies [18,25] for comparison.

Figure 9a shows the cell alignment characteristic of asymmetric comb structures containing a fixed SiO_2_ line width of 1 μm combined with varying trench widths. The results indicate that the cell alignment performance decreases on both types of substrates with increasing trench width as the line width is fixed. A similar trend in the reduced alignment of human corneal epithelial cells on monolithic SiO_2_ structures, containing parallel grooves 600 nm deep and ridges, was reported by Teixeira et al. [11]. They observed that the percentage of cells aligned to the pattern decreased from ~24% to ~5% when the ridge-to-groove width ratio increased from 1:1.1 to 1:4.7.

One possible explanation for the decrease in cell alignment performance on structures with trench widths, as shown in the results in Figure 9a, may be related to the shift in the mechanotransduction response of cells on different surface topographic features. Tsui et al. [58] cross-sectioned adherent Vero cells on monolithic Ta comb structures with equal trench and line widths. They observed adherent cells “float” on top of comb structures when trench and line widths are smaller than 0.25 μm. Almost none of the cellular materials extend into the trenches on these engineered surfaces. A noticeable change in cell response is observed on structures with trench and line widths between 0.5 μm and 2.0 μm; the majority of thin cellular components, such as filopodia and lamellipodia, located at the cell peripheries, extend into the trenches. However, the thick nuclei remain resting on top of the lines. For structures with 50 μm wide trenches and lines, Tsui’s cell cross-sectional studies have shown another change in cellular response, where the entire cell, including the nucleus, conformally covers the surface topographic structure. This appears to coincide with another shift in cell alignment performance on structures with a trench width of 50 μm, as shown in Figure 9a.

Cell alignment differences between the two types of substrates for each design pattern were also quantified, and their *p*-values are listed in Table 2. Test patterns with a *p*-value smaller than 0.05 indicate a statistically significant difference in cell alignment on the substrates. Figure 9a shows monolithic Ta surfaces (red solid circles) generally induced more cell alignment than the composite structures (blue squares), even on surfaces with the same topographic design. Figure 9a shows that introducing SiO_2_ lines did not enhance the capability to manipulate cells. When combining the two cell-manipulation approaches, an opposite effect is observed—a decrease in the likelihood of cells aligning parallel to the line axes. More importantly, the results in Figure 9a indicate that the dependence of cell alignment on trench width can be divided into two distinct regions: (i) from 1 μm to 9 μm and (ii) from 9 μm to 50 μm. In region (i), the degree of degradation decreases with trench width. At a trench width of 5 μm and 9 μm, there is no statistically significant difference in cell alignment performance between the two types of substrates. Our results are believed to be the first report demonstrating that these two cell manipulation mechanisms may not complement each other and that the degree of impact varies with pattern design.

The performance of cell alignment on inverted patterns with a fixed Ta trench width of 1 μm is shown in Figure 9b. This figure indicates that cell orientation becomes more disorganized as line widths increase, regardless of the substrate composition. Interestingly, the degradation in cell alignment caused by SiO_2_ lines was only considered statistically significant on substrates with 1 μm line widths; no significant degradation was observed in other patterns of the remaining fixed Ta trench width group (see Table 2 for the *p*-values). This evidence suggests that cell alignment degradation due to the introduction of SiO_2_ lines depends on the layout of the topographic features. A similar decrease in cell (HeLa) alignment performance on a microgroove polydimethylsiloxane (PDMS) surface, featuring a fixed groove width (2 mm) and variable ridge width topographic structure, was reported by Zhou et al. [59]. They showed that the population of cell alignment decreased from ~83% to ~70% when the groove-to-ridges width dimension increased from 1 μm–2 μm to 2 μm–30 μm.

The results presented in Figure 9 show that the comb pattern and substrate composition influence the degradation in cell alignment performance. While substrates with either fixed trench width or fixed line width can be used to manipulate cells, the former appears less sensitive to the degradation induced by the presence of SiO_2_ lines. This information is valuable for designing engineered surfaces that induce specific cell or tissue morphology. The specific origin of this SiO_2_ line-induced alignment decay has yet to be fully understood. However, it is well established that external factors can cause both genes and/or proteins to be either up-regulated or down-regulated, altering the cell’s molecular pathways. For instance, Bencharit et al. [60] found that exposure to a porous Ta implant, as opposed to a titanium alloy control sample, led to the up-regulation of several bone morphogenic proteins and collagens. Leven et al. investigated the effects of surface roughness (0.14 μm and 5.8 μm) on rat bone marrow stromal cells gene expression on Ti_6_Al_4_V substrate surfaces [61]. They discovered that cells incubated on 5.8 μm surfaces exhibited a down-regulation of laminin but an up-regulation of vitronectin, integrin α1, and fibronectin. Similar changes were observed in other bone gene expressions (osteonectin and alkaline phosphatase) and ECM gene expressions (collagen molecules). Their results suggest that the impact of substrate material composition and topography is intricate. The topography-driven mechanism is the material-induced up-regulation of a specific protein, or vice versa.

It is beyond the scope of the current work to analyze some of the substrate design components that influence the molecular-level impacts of these comb structures. However, this work demonstrates that understanding the overall impact of cell interaction with composite substrates requires a comprehensive approach to understanding the effects of topography and substrate material composition. It is important to note that the focus of this work is limited to cell behaviors when the coverage is less than one monolayer, and cells are incubated for only 24 h. The cell alignment performance may change if the cell coverage involves multiple layers of cells, the cell concentration increases, or the incubation time exceeds 24 h. Future studies should investigate the biophysical mechanisms underlying how and why SiO_2_ affects cell alignment performance. A question remains about which cell adhesive protein adsorbs preferentially on Ta. Additional focus on the impact of surface energy or surface chemistry on protein adsorption and focal adhesion should be studied.

## 4. Conclusions

This study examines how mammalian cells behave on monolithic tantalum (Ta) and silicon oxide (SiO_2_) asymmetric comb structures. We found that three design parameters for the surface influenced how cells behaved: the width of the trenches, the width of the lines, and the choice of material. The results indicate that the highest alignment on both structures (monolithic and composite) happened when both the trenches and lines were 1 μm wide. The results also indicate that the alignment decreased with increasing line or trench width. Additionally, replacing part of the Ta line surface with SiO_2_ on the Ta comb structure reduced control over cell alignment. The mechanisms for controlling cell behavior with surface topography and different materials in the Ta/SiO_2_ system did not act synergistically; rather, there appears to exist a complex interplay between these parameters that demands further investigation. They negated the benefits of each mechanism.

## Figures and Tables

**Table 1:** The percentage population of cells aligned within ±10° from the line axes on two substrate types consisted of comb structures with various combinations of trench and line widths. Data spread corresponds to one standard deviation. Cell alignment results from symmetric comb structures with equal trench and line widths of 1 μm are included as a comparison [18,25]. The number of cells inspected on each comb structure (n) is also included in this table.

Specimen Number	Substrate Surface Material	Width (μm)		Total Cell Count (n)	% Cell Aligned ±10° from Line Axes
		Trench	Line		
1	Ta	1	1	197 [18]	78 ± 2 [18]
2	Ta	3	1	184	61 ± 7
3	Ta	5	1	241	43 ± 5
4	Ta	9	1	275	28 ± 4
5	Ta	50	1	158	31 ± 3
6	Ta	1	3	219	46 ± 7
7	Ta	1	5	201	40 ± 3
8	Ta	1	9	198	42 ± 6
9	Ta	1	50	197	24 ± 3
10	Ta/SiO_2_	1	1	262 [25]	63 ± 1 [25]
11	Ta/SiO_2_	1	3	282	44 ± 5
12	Ta/SiO_2_	1	5	282	40 ± 5
13	Ta/SiO_2_	1	9	346	31 ± 4
14	Ta/SiO_2_	1	50	271	29 ± 7
15	Ta/SiO_2_	3	1	289	38 ± 6
16	Ta/SiO_2_	5	1	370	34 ± 2
17	Ta/SiO_2_	9	1	358	32 ± 6
18	Ta/SiO_2_	50	1	308	18 ± 1

**Table 2:** List of *p*-values from cell alignment comparisons between two types of substrates: one with a monolithic tantalum surface and the other with a tantalum/SiO_2_ composite surface. The test patterns have fixed trench or line widths of 1 μm. Test patterns with a *p*-value smaller than 0.05 indicate a statistically significant difference.

Trench Width (μm)	Line Width (μm)	*p*-Value	Significant Different
1	1	0.003	Yes
1	3	0.688	No
1	5	0.941	No
1	9	0.058	No
1	50	0.367	No
1	1	0.003	Yes
3	1	0.013	Yes
5	1	0.052	No
9	1	0.463	No
50	1	0.006	Yes

## Data Availability

The original contributions presented in this study are included in the article. Further inquiries can be directed to the corresponding author.
